# (*E*)-4-Methyl-2-{[tris(hydroxymethyl)methyl]iminiomethyl}phenolate

**DOI:** 10.1107/S1600536809051800

**Published:** 2009-12-09

**Authors:** Gonca Özdemir Tarı, Hasan Tanak, Mustafa Macit, Ferda Erşahin, Şamil Isık

**Affiliations:** a Department of Physics, Faculty of Arts & Science, Ondokuz Mayıs University, TR-55139, Kurupelit-Samsun, Turkey; b Department of Chemistry, Faculty of Arts & Science, Ondokuz Mayıs University, 55139 Samsun, Turkey

## Abstract

In the zwitterionic title compound, C_12_H_17_NO_4_, an intra­molecular N—H⋯O hydrogen bond generates a six-membered ring, producing an *S*(6) ring. In the crystal structure, mol­ecules are linked by inter­molecular C—H⋯O and O—H⋯O inter­actions.

## Related literature

For the properties and uses of Schiff bases, see: Aydoğan *et al.* (2001 ▶); Barton & Ollis (1979 ▶); Layer (1963 ▶); Ingold (1969 ▶); Cohen *et al.* (1964 ▶); Ogawa & Harada (2003 ▶); Taggi *et al.* (2002 ▶). For hydrogen-bond motifs, see: Bernstein *et al.* (1995 ▶). For comparative bond lengths, see: Allen *et al.* (1987 ▶); Yüce *et al.* (2006 ▶).
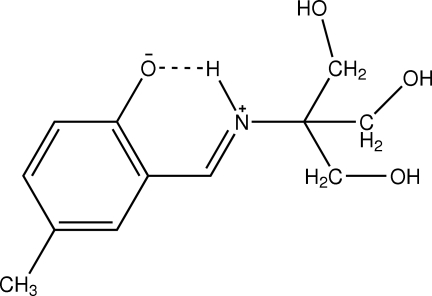


## Experimental

### 

#### Crystal data

C_12_H_17_NO_4_
*M* *_r_* = 239.27
Triclinic, 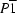

*a* = 6.7501 (6) Å
*b* = 8.5036 (8) Å
*c* = 11.129 (1) Å
α = 87.584 (8)°
β = 77.192 (8)°
γ = 79.215 (8)°
*V* = 611.9 (1) Å^3^
*Z* = 2
Mo *K*α radiation
μ = 0.10 mm^−1^
*T* = 296 K
0.80 × 0.48 × 0.21 mm

#### Data collection

STOE IPDS II diffractometer
Absorption correction: integration (*X-RED32*; Stoe & Cie, 2002 ▶) *T*_min_ = 0.942, *T*_max_ = 0.979
8321 measured reflections
2409 independent reflections
2001 reflections with *I* > 2σ(*I*)
*R*_int_ = 0.022

#### Refinement

*R*[*F* ^2^ > 2σ(*F* ^2^)] = 0.036
*wR*(*F* ^2^) = 0.105
*S* = 1.09
2409 reflections
160 parameters
H atoms treated by a mixture of independent and constrained refinement
Δρ_max_ = 0.25 e Å^−3^
Δρ_min_ = −0.22 e Å^−3^

### 

Data collection: *X-AREA* (Stoe & Cie, 2002 ▶); cell refinement: *X-AREA*; data reduction: *X-RED32* (Stoe & Cie, 2002 ▶); program(s) used to solve structure: *SHELXS97* (Sheldrick, 2008 ▶); program(s) used to refine structure: *SHELXL97* (Sheldrick, 2008 ▶); molecular graphics: *ORTEP-3 for Windows* (Farrugia, 1997 ▶); software used to prepare material for publication: *WinGX* (Farrugia, 1999 ▶).

## Supplementary Material

Crystal structure: contains datablocks I. DOI: 10.1107/S1600536809051800/im2160sup1.cif

Structure factors: contains datablocks I. DOI: 10.1107/S1600536809051800/im2160Isup2.hkl

Additional supplementary materials:  crystallographic information; 3D view; checkCIF report

## Figures and Tables

**Table 1:** Hydrogen-bond geometry (Å, °)

*D*—H⋯*A*	*D*—H	H⋯*A*	*D*⋯*A*	*D*—H⋯*A*
O4—H4⋯O1^i^	0.82	1.89	2.621 (1)	148
O3—H13⋯O1^ii^	0.82	1.86	2.679 (1)	178
O2—H14⋯O4^iii^	0.82	2.03	2.821 (1)	163
C8—H8⋯O3^iv^	0.93	2.35	3.272 (2)	171
C10—H10*A*⋯O3^iv^	0.97	2.58	3.379 (2)	140
N1—H1⋯O1	0.86 (2)	1.93 (2)	2.638 (1)	138 (1)

Symmetry codes: (i) 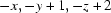; (ii) 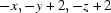; (iii) 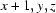; (iv) 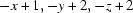.

**Fig. 1.**
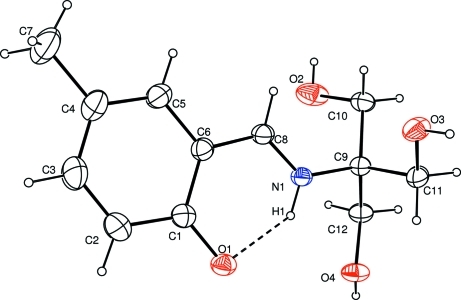
The molecular structure of the title compound, showing the atom-numbering scheme and 30% probability diplacement ellipsoids.

**Fig. 2.**
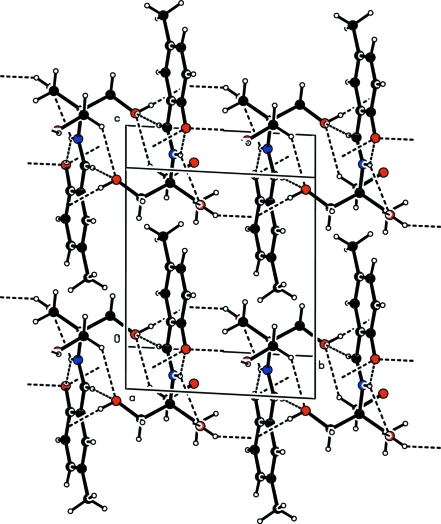
The crystal packing of the title compound. Intra- and intermolecular hydrogen bonds are shown as dashed lines.

## supplementary crystallographic information

### Comment

Schiff bases are used as starting materials in the synthesis of important drugs, such as antibiotics and antiallergic, antiphlogistic, and antitumor substances (Barton *et al.*, 1979; Layer, 1963; Ingold 1969). On the industrial scale, they have a wide range of applications, such as dyes and pigments (Taggi *et al.*, 2002). Schiff bases have also been employed as ligands for the complexation of metal ions (Aydoğan *et al.*, 2001). There are two characteristic properties of Schiff bases, *viz*. photochromism and thermochromism (Cohen *et al.*, 1964). In general, Schiff bases based on salicylic alehyde display two possible tautomeric forms, the iminomethyl-phenol (OH) and the aminomethylene-cyclohexa-2,4-dienone (NH) forms. Depending on the tautomers, two types of intramolecular hydrogen bonds are observed in Schiff bases: O—H···N in the former and N—H···O in the latter tautomer. Another form of the Schiff base compounds is also regarded to be zwitterionic showing an ionic intramolecular hydrogen bond (N^+^—H···O^-^) and this form is rarely seen in the solid state. The NH form of Schiff bases in the solid state can be regarded as a resonance hybrid of two canonical structures, the aminomethylene-cyclohexa-2,4-dienone and the zwitterionic form (Ogawa, *et al.*,2003).

The crystal and molecular structure of the title compound, C_12_H_17_NO_4_, has been synthesized and *x*-ray single-crystal structure determination has been performed. The title molecule exists in a zwitterionic form with a strong intramolecular N^+^—H···O^-^ hydrogen bond between the NH^+^ and the phenolate O^-^. The bond lengths and angles are within normal ranges (Allen *et al.*,1987). The C8=N1 [1.286 (2) Å] and C1—O1 [1.307 (2) Å] bonds may be compared with the corresponding values [1.295 (2) and 1.295 (2) Å] in a similar zwitterionic structure (Yüce *et al.*, 2006). Nevertheless, carbon carbon bonds in the phenyl group are slightly alternating reminding of the aminomethylene-cyclohexa-2,4-dienone canonical structure.

As it is expected, the salicylaldimine subunit of the molecule is almost perfectly planar whereas C9 as a sp^3^ hybridised carbon is tetrahedrally coordinated (Fig. 1). Torsion angles C8—N1—C9—C10, C8—N1—C9—C11 and C8—N1—C9—C12 are -20.3 (2)°, 100.8 (1)° and -140.9 (1)°, respectively. Bond lengths and angles in the planar salicylaldimine fragment and in the C(CH_2_OH)_3_ group of the studied compound are in a good agreement with the related compounds (Allen *et al.*, 1987; Yüce *et al.*, 2006).

An intramolecular N1—H1···O1 hydrogen bond generates a six-membered ring, producing an S(6) ring motif (Bernstein *et al.*, 1995). In the crystal structure, molecules are linked together by intermolecular C—H···O and O—H···O interactions (Fig. 2).

### Experimental

The title compound (*E*)-2-hydroxymethyl-2-[(2-oxo-5-methyl-benzylidene) -ammonium]-propane-1,3-diol was prepared by refluxing a mixture of 5-methylsalicylaldehyde (0.05 g 0.36 mmol) and tris(hydroxymethyl)aminomethane (0.0435 g 0.25 mmol) in 40 ml ethanol for one hour. Crystals of the title compound suitable for *x*-ray analysis were obtained from n-hexane/methanol (1:1) by slow evaporation (yield 88%; m.p. 429–430 K).

### Refinement

The position of H1 was obtained from a difference Fourier map and was refined freely. Other H atoms were positioned geometrically and treated using a riding model, fixing the bond lengths at 0.82 Å for OH, at 0.93 Å for aromatic CH, at 0.96 Å for CH_3_, at 0.97 Å for CH_2_. The displacement parameters of the H atoms were constrained as *U*_iso_(H)= 1.2*U*_eq_ (1.5*U*_eq_ for methyl) of the parent atom.

### Crystal data

**Table:**

C_12_H_17_NO_4_	*Z* = 2
*M**_r_* = 239.27	*F*(000) = 256
Triclinic, *P*1	*D*_x_ = 1.299 Mg m^−^^3^
Hall symbol: -P 1	Mo *K*α radiation, λ = 0.71073 Å
*a* = 6.7501 (6) Å	Cell parameters from 13032 reflections
*b* = 8.5036 (8) Å	θ = 1.9–28.0°
*c* = 11.129 (1) Å	µ = 0.10 mm^−^^1^
α = 87.584 (8)°	*T* = 296 K
β = 77.192 (8)°	PRISM., yellow
γ = 79.215 (8)°	0.80 × 0.48 × 0.21 mm
*V* = 611.9 (1) Å^3^	

### Data collection

**Table:**

STOE IPDS II diffractometer	2409 independent reflections
Radiation source: fine-focus sealed tube	2001 reflections with *I* > 2σ(*I*)
graphite	*R*_int_ = 0.022
Detector resolution: 6.67 pixels mm^-1^	θ_max_ = 26.0°, θ_min_ = 1.9°
rotation method scans	*h* = −8→8
Absorption correction: integration (*X-RED32*; Stoe & Cie, 2002)	*k* = −10→10
*T*_min_ = 0.942, *T*_max_ = 0.979	*l* = −13→13
8321 measured reflections	

### Refinement

**Table:**

Refinement on *F*^2^	Secondary atom site location: difference Fourier map
Least-squares matrix: full	Hydrogen site location: inferred from neighbouring sites
*R*[*F*^2^ > 2σ(*F*^2^)] = 0.036	H atoms treated by a mixture of independent and constrained refinement
*wR*(*F*^2^) = 0.105	*w* = 1/[σ^2^(*F*_o_^2^) + (0.0534*P*)^2^ + 0.0953*P*] where *P* = (*F*_o_^2^ + 2*F*_c_^2^)/3
*S* = 1.09	(Δ/σ)_max_ < 0.001
2409 reflections	Δρ_max_ = 0.25 e Å^−^^3^
160 parameters	Δρ_min_ = −0.21 e Å^−^^3^
0 restraints	Extinction correction: *SHELXL*, Fc^*^=kFc[1+0.001xFc^2^λ^3^/sin(2θ)]^-1/4^
Primary atom site location: structure-invariant direct methods	Extinction coefficient: 0.046 (8)

### Special details

**Table:**

Experimental. 339 frames, detector distance = 100 mm
Geometry. All e.s.d.'s (except the e.s.d. in the dihedral angle between two l.s. planes) are estimated using the full covariance matrix. The cell e.s.d.'s are taken into account individually in the estimation of e.s.d.'s in distances, angles and torsion angles; correlations between e.s.d.'s in cell parameters are only used when they are defined by crystal symmetry. An approximate (isotropic) treatment of cell e.s.d.'s is used for estimating e.s.d.'s involving l.s. planes.
Refinement. Refinement of *F*^2^ against ALL reflections. The weighted *R*-factor *wR* and goodness of fit *S* are based on *F*^2^, conventional *R*-factors *R* are based on *F*, with *F* set to zero for negative *F*^2^. The threshold expression of *F*^2^ > σ(*F*^2^) is used only for calculating *R*-factors(gt) *etc*. and is not relevant to the choice of reflections for refinement. *R*-factors based on *F*^2^ are statistically about twice as large as those based on *F*, and *R*- factors based on ALL data will be even larger.

### Fractional atomic coordinates and isotropic or equivalent isotropic displacement parameters (Å^2^)

**Table:**

	*x*	*y*	*z*	*U*_iso_*/*U*_eq_	
C1	0.2299 (2)	0.70721 (15)	0.73471 (12)	0.0388 (3)	
C2	0.2401 (3)	0.6812 (2)	0.60930 (15)	0.0587 (4)	
H2	0.1327	0.6440	0.5865	0.070*	
C3	0.4045 (3)	0.7094 (3)	0.52063 (15)	0.0667 (5)	
H3	0.4062	0.6888	0.4390	0.080*	
C4	0.5709 (3)	0.7680 (2)	0.54645 (14)	0.0570 (4)	
C5	0.5658 (2)	0.79155 (18)	0.66829 (13)	0.0476 (4)	
H5	0.6752	0.8284	0.6891	0.057*	
C6	0.4002 (2)	0.76175 (15)	0.76301 (12)	0.0377 (3)	
C7	0.7462 (3)	0.8029 (3)	0.44497 (17)	0.0820 (6)	
H7A	0.8509	0.8331	0.4804	0.123*	
H7B	0.6954	0.8887	0.3946	0.123*	
H7C	0.8040	0.7089	0.3952	0.123*	
C8	0.4134 (2)	0.78295 (15)	0.88719 (12)	0.0364 (3)	
H8	0.5246	0.8244	0.9009	0.044*	
C9	0.27900 (19)	0.76614 (14)	1.11183 (11)	0.0333 (3)	
C10	0.4917 (2)	0.77744 (17)	1.13130 (13)	0.0422 (3)	
H10A	0.5351	0.8725	1.0910	0.051*	
H10B	0.4869	0.7853	1.2186	0.051*	
C11	0.1251 (2)	0.91827 (15)	1.15916 (13)	0.0409 (3)	
H11A	−0.0106	0.9114	1.1465	0.049*	
H11B	0.1148	0.9307	1.2467	0.049*	
C12	0.2064 (2)	0.61933 (16)	1.17826 (13)	0.0395 (3)	
H12A	0.3071	0.5238	1.1501	0.047*	
H12B	0.1934	0.6296	1.2663	0.047*	
N1	0.28000 (16)	0.74792 (12)	0.98133 (9)	0.0325 (3)	
O1	0.07182 (14)	0.68314 (10)	0.82072 (9)	0.0412 (3)	
O2	0.63352 (16)	0.64022 (13)	1.08193 (13)	0.0655 (4)	
H14	0.7485	0.6460	1.0922	0.098*	
O3	0.19503 (15)	1.05030 (11)	1.09428 (10)	0.0510 (3)	
H13	0.1139	1.1327	1.1190	0.077*	
O4	0.01450 (14)	0.60604 (11)	1.15400 (9)	0.0452 (3)	
H4	−0.0244	0.5273	1.1897	0.068*	
H1	0.179 (3)	0.7122 (19)	0.9641 (14)	0.047 (4)*	

### Atomic displacement parameters (Å^2^)

**Table:**

	*U*^11^	*U*^22^	*U*^33^	*U*^12^	*U*^13^	*U*^23^
C1	0.0392 (7)	0.0321 (6)	0.0448 (7)	−0.0052 (5)	−0.0094 (6)	0.0000 (5)
C2	0.0602 (10)	0.0732 (11)	0.0495 (9)	−0.0187 (8)	−0.0198 (8)	−0.0046 (8)
C3	0.0721 (12)	0.0905 (13)	0.0386 (8)	−0.0158 (10)	−0.0134 (8)	−0.0033 (8)
C4	0.0544 (10)	0.0684 (10)	0.0420 (8)	−0.0070 (8)	−0.0013 (7)	0.0030 (7)
C5	0.0414 (8)	0.0553 (8)	0.0445 (8)	−0.0127 (6)	−0.0033 (6)	0.0017 (6)
C6	0.0381 (7)	0.0357 (6)	0.0382 (7)	−0.0079 (5)	−0.0045 (5)	−0.0010 (5)
C7	0.0720 (13)	0.1168 (18)	0.0473 (10)	−0.0174 (12)	0.0065 (9)	0.0066 (10)
C8	0.0331 (6)	0.0340 (6)	0.0426 (7)	−0.0099 (5)	−0.0056 (5)	−0.0014 (5)
C9	0.0315 (6)	0.0337 (6)	0.0355 (6)	−0.0097 (5)	−0.0060 (5)	0.0001 (5)
C10	0.0380 (7)	0.0470 (7)	0.0461 (7)	−0.0147 (6)	−0.0133 (6)	0.0016 (6)
C11	0.0389 (7)	0.0360 (7)	0.0450 (7)	−0.0114 (5)	0.0017 (6)	−0.0042 (5)
C12	0.0370 (7)	0.0382 (7)	0.0457 (7)	−0.0126 (5)	−0.0109 (6)	0.0078 (5)
N1	0.0289 (5)	0.0316 (5)	0.0380 (6)	−0.0087 (4)	−0.0063 (4)	−0.0013 (4)
O1	0.0373 (5)	0.0354 (5)	0.0522 (6)	−0.0108 (4)	−0.0086 (4)	−0.0016 (4)
O2	0.0382 (6)	0.0497 (6)	0.1130 (10)	−0.0030 (5)	−0.0300 (6)	−0.0023 (6)
O3	0.0417 (6)	0.0313 (5)	0.0726 (7)	−0.0088 (4)	0.0055 (5)	−0.0010 (4)
O4	0.0361 (5)	0.0378 (5)	0.0659 (7)	−0.0156 (4)	−0.0140 (4)	0.0094 (4)

### Geometric parameters (Å, °)

**Table:**

C1—O1	1.3071 (16)	C9—N1	1.4654 (16)
C1—C2	1.407 (2)	C9—C10	1.5197 (17)
C1—C6	1.4174 (19)	C9—C12	1.5282 (17)
C2—C3	1.363 (3)	C9—C11	1.5305 (18)
C2—H2	0.9300	C10—O2	1.4080 (19)
C3—C4	1.399 (3)	C10—H10A	0.9700
C3—H3	0.9300	C10—H10B	0.9700
C4—C5	1.371 (2)	C11—O3	1.4085 (16)
C4—C7	1.508 (2)	C11—H11A	0.9700
C5—C6	1.4094 (19)	C11—H11B	0.9700
C5—H5	0.9300	C12—O4	1.4060 (16)
C6—C8	1.4255 (18)	C12—H12A	0.9700
C7—H7A	0.9600	C12—H12B	0.9700
C7—H7B	0.9600	N1—H1	0.858 (18)
C7—H7C	0.9600	O2—H14	0.8200
C8—N1	1.2856 (16)	O3—H13	0.8200
C8—H8	0.9300	O4—H4	0.8200
			
O1—C1—C2	122.02 (13)	C10—C9—C12	110.25 (10)
O1—C1—C6	121.62 (12)	N1—C9—C11	107.87 (10)
C2—C1—C6	116.36 (13)	C10—C9—C11	109.93 (11)
C3—C2—C1	121.25 (15)	C12—C9—C11	110.19 (11)
C3—C2—H2	119.4	O2—C10—C9	109.24 (11)
C1—C2—H2	119.4	O2—C10—H10A	109.8
C2—C3—C4	123.19 (15)	C9—C10—H10A	109.8
C2—C3—H3	118.4	O2—C10—H10B	109.8
C4—C3—H3	118.4	C9—C10—H10B	109.8
C5—C4—C3	116.52 (15)	H10A—C10—H10B	108.3
C5—C4—C7	122.08 (16)	O3—C11—C9	108.51 (10)
C3—C4—C7	121.40 (16)	O3—C11—H11A	110.0
C4—C5—C6	122.10 (14)	C9—C11—H11A	110.0
C4—C5—H5	118.9	O3—C11—H11B	110.0
C6—C5—H5	118.9	C9—C11—H11B	110.0
C5—C6—C1	120.53 (12)	H11A—C11—H11B	108.4
C5—C6—C8	117.94 (12)	O4—C12—C9	109.49 (10)
C1—C6—C8	121.50 (12)	O4—C12—H12A	109.8
C4—C7—H7A	109.5	C9—C12—H12A	109.8
C4—C7—H7B	109.5	O4—C12—H12B	109.8
H7A—C7—H7B	109.5	C9—C12—H12B	109.8
C4—C7—H7C	109.5	H12A—C12—H12B	108.2
H7A—C7—H7C	109.5	C8—N1—C9	127.73 (11)
H7B—C7—H7C	109.5	C8—N1—H1	114.8 (10)
N1—C8—C6	123.60 (12)	C9—N1—H1	117.5 (10)
N1—C8—H8	118.2	C10—O2—H14	109.5
C6—C8—H8	118.2	C11—O3—H13	109.5
N1—C9—C10	111.90 (10)	C12—O4—H4	109.5
N1—C9—C12	106.62 (10)		
			
O1—C1—C2—C3	178.89 (16)	C1—C6—C8—N1	3.4 (2)
C6—C1—C2—C3	−0.9 (2)	N1—C9—C10—O2	−57.33 (14)
C1—C2—C3—C4	−1.2 (3)	C12—C9—C10—O2	61.16 (15)
C2—C3—C4—C5	2.2 (3)	C11—C9—C10—O2	−177.17 (11)
C2—C3—C4—C7	−177.89 (19)	N1—C9—C11—O3	−62.87 (13)
C3—C4—C5—C6	−1.3 (2)	C10—C9—C11—O3	59.40 (14)
C7—C4—C5—C6	178.83 (16)	C12—C9—C11—O3	−178.89 (10)
C4—C5—C6—C1	−0.7 (2)	N1—C9—C12—O4	−55.27 (13)
C4—C5—C6—C8	177.23 (13)	C10—C9—C12—O4	−176.94 (11)
O1—C1—C6—C5	−178.02 (12)	C11—C9—C12—O4	61.54 (14)
C2—C1—C6—C5	1.7 (2)	C6—C8—N1—C9	−179.57 (11)
O1—C1—C6—C8	4.15 (19)	C10—C9—N1—C8	−20.27 (17)
C2—C1—C6—C8	−176.10 (13)	C12—C9—N1—C8	−140.89 (12)
C5—C6—C8—N1	−174.53 (13)	C11—C9—N1—C8	100.77 (14)

### Hydrogen-bond geometry (Å, °)

**Table:**

*D*—H···*A*	*D*—H	H···*A*	*D*···*A*	*D*—H···*A*
O4—H4···O1^i^	0.82	1.89	2.621 (1)	148
O3—H13···O1^ii^	0.82	1.86	2.679 (1)	178
O2—H14···O4^iii^	0.82	2.03	2.821 (1)	163
C8—H8···O3^iv^	0.93	2.35	3.272 (2)	171
C10—H10A···O3^iv^	0.97	2.58	3.379 (2)	140
N1—H1···O1	0.86 (2)	1.93 (2)	2.638 (1)	138 (1)

Symmetry codes: (i) −*x*, −*y*+1, −*z*+2; (ii) −*x*, −*y*+2, −*z*+2; (iii) *x*+1, *y*, *z*; (iv) −*x*+1, −*y*+2, −*z*+2.
